# Comparison of the Clinical Sensitivity of the Idylla Platform and the OncoBEAM RAS CRC Assay for KRAS Mutation Detection in Liquid Biopsy Samples

**DOI:** 10.1038/s41598-019-45616-y

**Published:** 2019-06-20

**Authors:** Ana Vivancos, Enrique Aranda, Manuel Benavides, Elena Élez, Maria Auxiliadora Gómez-España, Marta Toledano, Martina Alvarez, Maria Rosario Chica Parrado, Vanesa García-Barberán, Eduardo Diaz-Rubio

**Affiliations:** 1 Cancer Genomics Group, Vall d’Hebron Institute of Oncology, Barcelona, Spain; 2 Department of Medical Oncology, Reina Sofía University Hospital, CIBERONC, Córdoba, Spain; 3 Department of Medical Oncology, Hospital Universitario Regional y Virgen de la Victoria, Málaga, Spain; 4 Department of Medical Oncology Vall d’Hebron Institute of Oncology (CIBERONC), Barcelona, Spain; 5 IMIBIC Instituto Maimonides Investigación Biomédica de Córdoba, Córdoba, Spain; 6 Laboratorio de Biología Molecular del Cáncer. Centro de Investigaciones Médico Sanitarias, Universidad de Málaga, Málaga, Spain; 7 Laboratorio de Investigación Traslacional, IdISSC, Hospital Clínico San Carlos, CIBERONC, Madrid, Spain

**Keywords:** Gastrointestinal cancer, Cancer screening

## Abstract

*KRAS* mutations are common in colorectal cancer (CRC). In this setting, mutation status determination in circulating-free DNA from blood samples (liquid biopsy) has been shown to be a viable alternative to tissue testing. The objective of this study was to compare the sensitivity of two liquid biopsy methods for detecting *KRAS* mutations in plasma samples from metastatic CRC patients. Samples with a positive (KRAS-MUT+) result and a mutant allelic fraction (MAF) < 5% according to the OncoBEAM RAS CRC assay were pairly analyzed by the Idylla ctKRAS Mutation Test (n = 116). In a cohort of 71 patients with at least 1 year of follow-up, the progression-free survival (PFS) was determined according to MAF values. Idylla detected *KRAS* mutations in 81/116 OncoBEAM KRAS-MUT+ samples with MAF < 5% and in 48/79 samples with MAF < 1%. Concordance between OncoBEAM and Idylla significantly improved at higher MAF values. PFS rates at 6 and 12 months tended to be lower in patients with MAF levels between 1% and 5% than in those with levels <1%. OncoBEAM demonstrated greater sensitivity for plasma detection of *KRAS* mutations than Idylla. Importantly, our data identified a “gray zone” below 1% MAF where Idylla showed reduced *KRAS* mutation detection, highlighting the importance of an accurate method to provide the mutational status of CRC patients.

## Introduction

International guidelines recommend testing for hotspot mutations in the rat sarcoma viral oncogene homolog (*RAS*) gene family, including the kirsten RAS (*KRAS*) and neuroblastoma RAS (*NRAS*) proto-oncogenes, to exclude *RAS* mutation-positive metastatic colorectal cancer (mCRC) patients from receiving anti-epidermal growth factor receptor (EGFR) therapy^1,2^. This is because anti-EGFR agents do not provide meaningful survival benefits versus anti-angiogenic/chemotherapy regimens in mCRC patients whose tumors are not wild type (WT) with respect to *RAS* genes^3–6^. Accurate detection of *RAS* mutations in these patients is therefore of high clinical importance for therapy selection. Traditionally, formalin-fixed paraffin-embedded (FFPE) tumor samples have been used to determine the *RAS* mutation status of CRC patients in routine clinical practice. However, *RAS* status determination in circulating-free DNA from blood samples (known as liquid biopsy) has been shown to be a viable alternative to FFPE sample testing^7–11^. The use of liquid biopsy *RAS* mutation tests that lack the necessary sensitivity may lead to mischaracterization of mCRC patients as WT and in turn may diminish patient response to anti-EGFR therapy and lead to worse outcomes. Therefore, a direct comparison of available methods for *RAS* mutation status from liquid biopsy samples is warranted in order to evaluate the relative performance during routine clinical practice.

Several different platforms are available to perform blood-based *RAS* mutation analysis and each has varying levels of performance representative of the underlying test methodology. From an analytical standpoint, it is well established that digital PCR (dPCR) technologies inherently provide greater sensitivity than quantitative PCR (qPCR) techniques for somatic mutation detection^12,13^. The Idylla ctKRAS Mutation Test is a qPCR assay that has an analytical sensitivity of ≤1% for *KRAS* mutations in exons 2 and 3 and ≤5% for mutations in exon 4^14^. The OncoBEAM RAS CRC^15^ is a dPCR assay that detects mutations in both *KRAS* and *NRAS* oncogenes with an analytical sensitivity determined to be <0.02% mutant allelic fraction (MAF)^16^. The main goal of the present study was to evaluate the sensitivity of these two liquid biopsy methods for detecting *KRAS* mutations in plasma samples from mCRC patients, both of which detect mutations in codons 12, 13, 59, 61, 117 and 146 of the *KRAS* gene. In addition, we examined the clinical sensitivity of Idylla and OncoBEAM plasma *KRAS* mutation detection at <1% MAF by comparing plasma mutational analysis obtained by both platforms to those obtained by standard-of-care (SOC) FFPE tumor *RAS* testing on paired primary tumor tissue specimens.

## Results

### Patient cohort and samples for analysis

**Figure 1:**
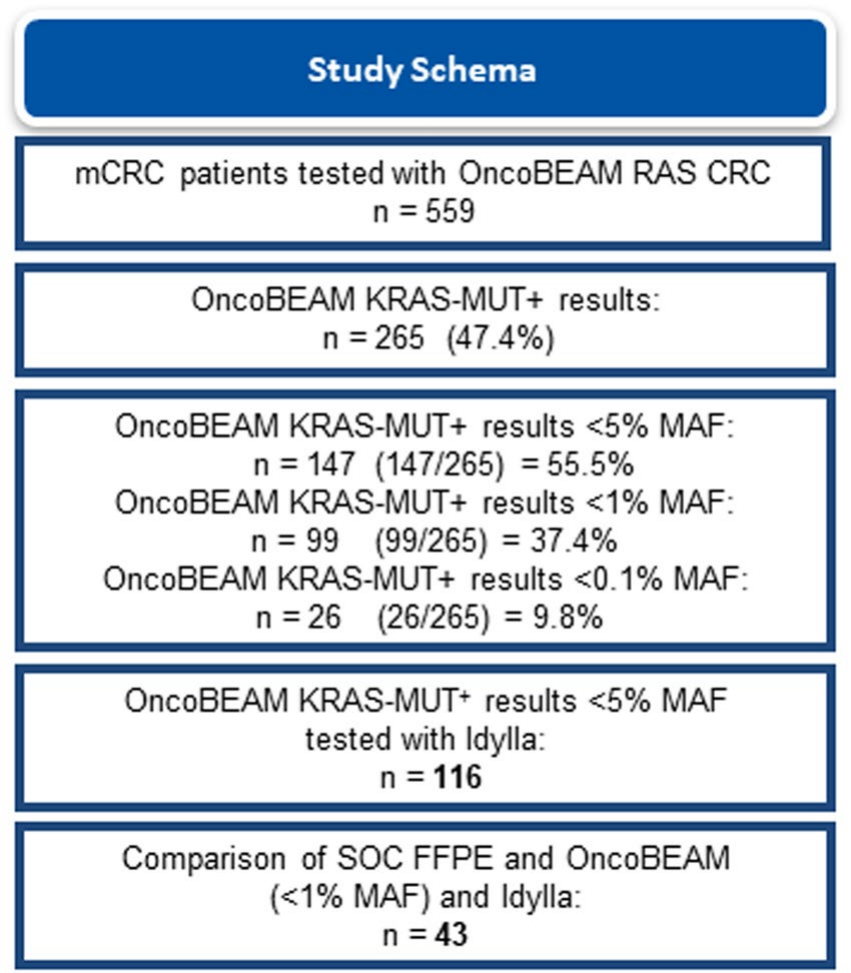
Flow chart of sample availability and analysis. Abbreviations: FFPE: formalin-fixed paraffin-embedded; MAF: mutant allele fraction; SOC: standard of care.

A total of 559 mCRC patients met the selection criteria. Median age was 66.9 years (range, 36–80) and the majority of them (97.3%) had colorectal adenocarcinoma. The most frequent site of metastasis was the liver (74.6%) followed by the lung (33.8%). Among the 559 plasma samples tested with the OncoBEAM RAS CRC assay, 265 (47.4%) were KRAS-MUT+, of which 147 (55.5%) had MAF < 5%. The 1-ml plasma requirement for Idylla testing could only be met for 116 out of the 147 samples, so the comparative analysis included only these 116 patients that were analyzed by the two liquid biopsy methods. SOC FFPE *KRAS* testing results on primary tumor specimens were only available for 43 KRAS-MUT+ patients with MAF values < 1% at the hospital centers performing OncoBEAM testing (Fig. 1).

### Concordance in mutation status between OncoBEAM and Idylla

The proportions of mutated samples at 3 different OncoBEAM-determined MAF% value ranges are shown in Table 1. Idylla detected *KRAS* mutations in 81 out of 116 OncoBEAM KRAS-MUT+ samples with MAF <5%, resulting in a PPA of 69.8%. Idylla detected *KRAS* mutations in 48 out of 79 OncoBEAM KRAS-MUT+ samples with MAF <1%, with a PPA of 60.7%. Concordance between OncoBEAM and Idylla significantly improved at higher MAF values.

**Table 1:** Comparison of Idylla and OncoBEAM KRAS mutation detection rates in plasma at different MAF %.

	Total	MAF < 0.1%	MAF 0.1% to <1%	MAF 1% to <5%	P-Value
N	116	16	63	37	
OncoBEAM Result: Mutated	116 (100.0%)	16 (100.0%)	63 (100.0%)	37 (100.0%)	1.0000 (a)
ldylla result: Mutated	81 (69.8%)	7 (43.8%)	41 (65.1%)	33 (89.2%)	**0.0005** (a)
p-value OncoBEAM vs ldylla (b)	**<0.0001**	**0.0077**	**<0.0001**	0.1336	

(a) Exact-Mantel-Haenszel; (b) paired McNemar Test. Abbreviations: MAF, mutated allele fraction.

### MAF and PFS

**Figure 2:**
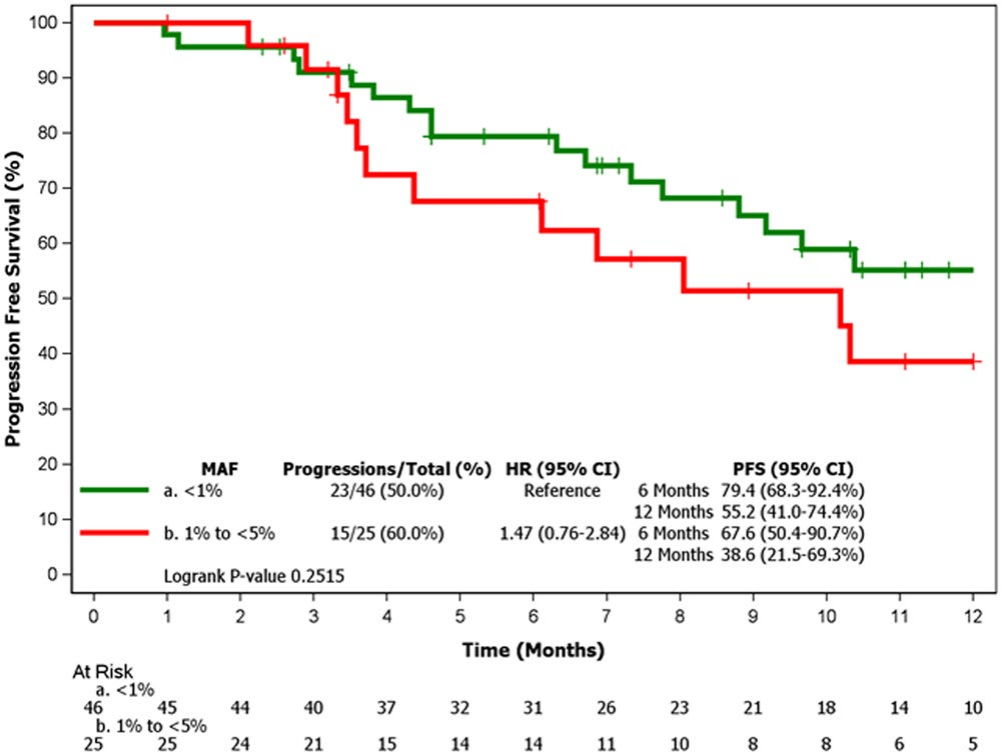
Kaplan-Meier curve of PFS according to MAF levels by BEAMing in plasma samples (n = 71). Abbreviations: CI: confidence interval; HR: hazard ratio; MAF: mutant allele fraction; PFS: progression-free survival.

PFS rates at 6 and 12 months were lower in patients with MAF levels between 1% and 5% than in those with levels <1% (Fig. 2), although the difference was not statistically significant (Hazard Ratio 1.47, 95% CI 0.76–2.84). Selected characteristics of patients with MAF < 1% and 1–5% are shown in Table 2.

**Table 2:** Patients characteristics according to MAF values determined by OncoBEAM assay.

	Total	MAF < 1%	MAF 1% to <5%	P-Value
**OncoBEAM Result**				
N	116	79	37	
Mutated	116 (100.0%)	79 (100.0%)	37 (100.0%)	1.0000 (a)
**Age (years)**				
N	70	46	24	
Median (Min/Max)	67.0 (35.6/80.1)	66.6 (35.6/79.3)	67.6 (36.3/80.1)	0.4254 (b)
**Sex**				
N	70	46	24	
Female	28 (40.0%)	19 (41.3%)	9 (37.5%)	0.8023 (a)
Male	42 (60.0%)	27 (58.7%)	15 (62.5%)	
**Months Since Initial Diagnostic Date**				
N	71	46	25	
Mediana (Min/Max)	3.2 (0.0/85.5)	3.8 (0.0/85.5)	1.9 (0.0/30.9)	0.0032 (b)
**Surgery**				
N	69	46	23	
Yes	42 (60.9%)	29 (63.0%)	13 (56.5%)	0.6119 (a)
**Histological Type**				
N	68	45	23	
Adenocarcinoma	67 (98.5%)	44 (97.8%)	23 (100.0%)	1.0000 (a)
**Months Since Metastasis Diagnostic Date**				
N	66	44	22	
Mediana (Min/Max)	1.6 (0.0/69.9)	1.6 (0.0/69.9)	1.5 (0.0/6.4)	0.0184 (b)
**Liver Metastasis**				
N	71	46	25	
Yes	53 (74.6%)	34 (73.9%)	19 (76.0%)	1.0000 (a)
**Lung Metastasis**				
N	71	46	25	
Yes	24 (33.8%)	13 (28.3%)	11 (44.0%)	0.1996 (a)
**Peritoneal Metastasis**				
N	71	46	25	
Yes	7 (9.9%)	5 (10.9%)	2 (8.0%)	1.0000 (a)
**Other Metastasis**				
N	71	46	25	
Yes	21 (29.6%)	15 (32.6%)	6 (24.0%)	0.5882 (a)
**Metastasis Surgery**				
N	71	46	25	
Yes	20 (28.2%)	15 (32.6%)	5 (20.0%)	0.2868 (a)
**Progression**				
N	71	46	25	
Yes	38 (53.5%)	23 (50.0%)	15 (60.0%)	0.4636 (a)

(a) Exact-Fisher; (b) Student T-test. Abbreviations: MAF, mutated allele fraction.

### Liquid biopsies versus tissue-based methods

**Figure 3:**
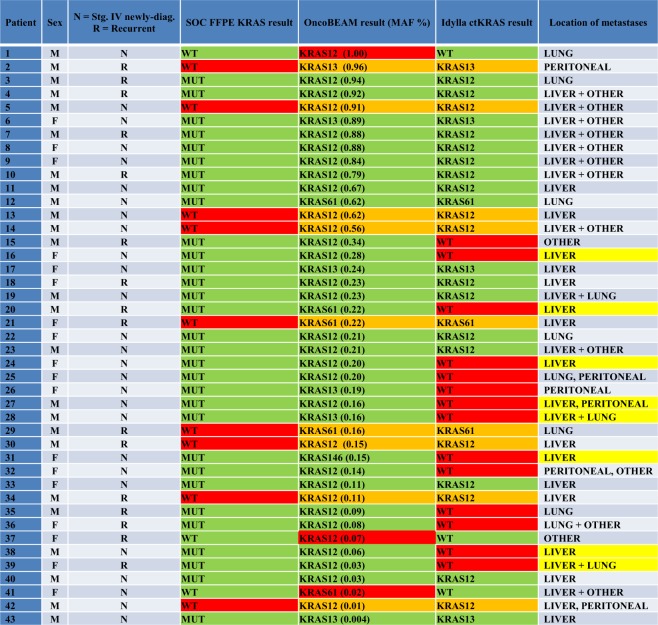
Descriptive summary of plasma and tissue mutation results (n = 43). Concordant and discordant results are highlighted in green and red, respectively. Discordance between Idylla vs OncoBEAM and SOC testing in patients with liver metastases are highlighted in yellow. Examples where both OncoBEAM and Idylla agree on KRAS-MUT+ call, but disagree with the SOC WT result are highlighted in orange. Abbreviations: F, female FFPE, formalin-fixed paraffin-embedded; M, male MAF, mutant allele fraction; MUT, mutated; SOC, standard of care; WT, wild-type.

**Figure 4:**
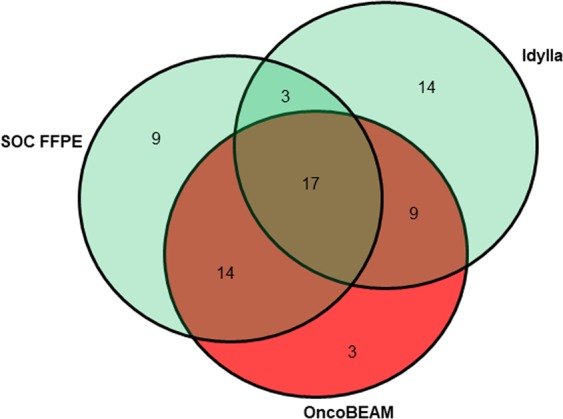
Venn diagram showing the numbers of *KRAS* mutated and wild-type samples according to the different technique used.

Idylla ctKRAS and OncoBEAM plasma testing results were compared with *KRAS* mutation results obtained by SOC FFPE tumor tissue testing in 43 mCRC patients that were OncoBEAM KRAS-MUT+ with MAF values <1%. The median time between tissue sampling and blood sampling was 247 days. The overall agreement (concordance) of OncoBEAM vs SOC FFPE *KRAS* testing results was 31/43 or 72.1%, whereas concordance of Idylla with SOC FFPE *KRAS* results was 20/43 or 46.5% (p = 0.0275). Idylla ctKRAS testing showed WT results in 14 out of 31 patients that were called KRAS-MUT+ by both OncoBEAM and SOC. Eight out of these 14 discordant cases were seen in patients with liver metastases (see Fig. 3 for further description of the mutation status determined by the three methods). Both Idylla and OncoBEAM calls were RAS-MUT+ in 9 out of 12 patients determined to be WT by tissue analysis (Fig. 4).

## Discussion

The accurate detection of hotspot mutations in the *KRAS* gene is integral to the diagnostic workup of mCRC patients to inform the appropriate use of targeted therapy^2^. A number of blood-based methods to detect *KRAS* mutations are currently available for clinical use^17^, though their head-to-head performance has previously not been directly compared. In the present study, results from the OncoBEAM RAS CRC mutation test were compared with the results obtained by the Idylla ctKRAS assay from paired plasma samples of mCRC patients analyzed by the two platforms. We only included those samples in which OncoBEAM had positive results (KRAS-MUT+) and MAF values were <5%, in order to examine the clinical sensibility at lower MAF. Patients with mCRC have been shown to exhibit a range of MAF values across newly diagnosed patients with primary tumor intact and those presenting with recurrent disease^7,18^. In cases with MAF < 1% by OncoBEAM, plasma mutational analysis was also compared to tissue-based methods on paired primary tumor specimens in order to independently examine the clinical sensitivity of Idylla and OncoBEAM plasma *KRAS* mutation detection.

The *KRAS* mutation frequency of 47.4% as determined by the OncoBEAM kit is in agreement with previously published reports of KRAS mutations in mCRC patients^6,7,19^. Overall, Idylla detected mutations in 69.8% of OncoBEAM KRAS-MUT+ samples, with higher concordance between the two methods at higher MAF levels. OncoBEAM has been shown to detect mutations as low as 0.02% MAF^16^, whereas the reported Idylla *KRAS* detection sensitivity is ≤1% MAF^14^. This means that most of the samples with low MAF analyzed by the Idylla ctKRAS kit will result in false-negative *KRAS* WT results and patients would inappropriately receive anti-EGFR therapy. In this scenario, the use of Idylla may incorrectly select patients for anti-EGFR therapy, exposing patients to undue side effects, increased medical costs, and worse outcomes due to lack of demonstrated clinical benefit of anti-EGFR therapy in patients with *RAS* mutations. Accordingly, the testing with Idylla ctKRAS resulted in a 16.9% reduction in clinical sensitivity for *KRAS* mutation detection vs SOC FFPE and OncoBEAM plasma testing. Since 37.4% of the 559 mCRC patients in this study were KRAS-MUT+ <1% MAF using OncoBEAM, it is estimated that a 16.9% drop in clinical sensitivity by Idylla might result in false negative calls for 45 out of 559 patients in this study. Even in cases of liver metastases, supposed to have greater shedding properties due to high vascularization^19,20^, Idylla was not able to detect *KRAS* mutations in 8 of 22 patients with tissue mutated status.

While OncoBEAM exhibited superior clinical sensitivity when compared to Idylla on replicate samples across MAF values, 12 out of 43 patients that were KRAS-MUT+ by OncoBEAM at <1% MAF were WT by SOC FFPE tissue testing. Our initial suspicion was that these low MAF samples might be false positives determined by OncoBEAM, but 9 out of 12 were also determined to be KRAS-MUT+ by Idylla. Although these data represent a small subset, it lends support to the conclusion that some of these patients may in fact be KRAS-MUT+ and were missed by SOC tissue testing either due to molecular heterogeneity or an insufficiency of the tissue mutation method. Testing of DNA from a single primary tumor tissue block may wrongly assign *KRAS* WT status in 8–11.6% of patients^21,22^. When inter-tumor heterogeneity between primary tumors and metastases is examined there is frequent mutational discordance^23,24^, and tumor genomic evolution likely occurring between tissue and blood sampling can also contribute to discordant results. Thus, the use of liquid biopsy methods is a valuable alternative to tissue-based testing to identify patients eligible for anti-EGFR therapy in routine clinical practice, avoiding the sample bias associated with intra- and inter-tumor heterogeneity. In addition, serial circulating tumor DNA (ctDNA) analysis enables monitoring of genomic changes during therapy and profiling of the global status of genomic alterations across different sites of disease^25^.

Testing for extended *RAS* mutation status is now vital component of routine standard of care workup for newly diagnosed mCRC patients, which includes hotspots regions of both *KRAS* and *NRAS* genes^2,26^. Since approximately 50% of the tested samples will return a WT *KRAS* result, these samples should also be tested for *NRAS* mutations. Therefore, it is valuable an extended *RAS* test to detect not only *KRAS* mutations but also *NRAS* mutations. The OncoBEAM RAS CRC kit evaluates both *KRAS* and *NRAS* mutations concurrently whereas Idylla testing for *KRAS* and *NRAS* is split into two different test runs to be performed sequentially. For both methods, the incorporation of the *RAS* mutational status in the initial histological report is now possible, enabling rapid initiation of targeted therapy and global assessment of tumor mutation status in newly diagnosed metastatic patients. An advantage of the BEAMing technology is its quantitative nature and high sensitivity. It has been shown that high *RAS* ctDNA MAF is associated with low survival^27^, so ctDNA levels as reflection of tumor load could provide valuable information to predict the disease evolution in *RAS* mutant patients prior to, during, or following treatment^28,29^.

This concept of MAF as a prognostic tool is consistent with our prior investigations and supported by the observation that patients with higher abundant *KRAS* mutations (MAF ≥ 1%) had a shorter PFS compared with those carrying low abundant *KRAS* mutations (MAF < 1%)^19^. Moreover, rapid progressors in our study had significantly higher MAF than slower progressors. This information can be readily incorporated to inform the clinician of those patients that may be “high risk” and require more intense treatment and radiological follow-up. As ctDNA can reveal occult metastatic disease that is not evident on radiological imaging^30^, we are now able to identify CRC patients at high risk of metastatic relapse and/or local recurrence, which is consistent with studies showing that ctDNA can signal residual disease after surgical resection^31,32^. It is unlikely that a single time-point following surgery will identify all patients who are going to relapse, but longitudinal sampling may resolve this issue and improve the sensitivity^30^. Moreover, WT patients that do receive anti-EGFR therapy can be monitored during treatment to detect acquired resistance due to *RAS* mutation emergence^19,29,33^.

Certain limitations exist in our study. One is the retrospective nature of the study and the lack of clinical information in a substantial proportion of patients, including the treatment they received and its potential effect on MAF values. Second, the small number of available tissue samples hindered a consistent comparison of concordance between plasma-based and tissue-based methods. Third, our data were limited to *KRAS* mutations, while we are now at the point where the technology should detect all *RAS* mutations beyond *KRAS* mutations, which is not the case for this Idylla kit. Finally, since there were no wild-type samples there was no evaluation of specificity, out of the scope of the study.

## Conclusion

Given that the frequency of mutant DNA alleles in cell-free DNA is as low as 0.01%^34^, highly sensitive and specific detection methods are required for ctDNA to deliver a clinically feasible and accurate approach. In this study, OncoBEAM demonstrated greater sensitivity for plasma detection of *KRAS* mutations than Idylla. Importantly, our data identified a “gray zone” below 1% MAF where Idylla shows reduced *KRAS* mutation detection accuracy vs OncoBEAM and SOC FFPE tumor *KRAS* testing. These findings serve as a reminder that liquid biopsy assays with diminished sensitivity may lack the dynamic range to provide the accurate mutational status to properly guide individualized anti-EGFR treatment decisions and risk stratification that may benefit patient outcomes.

## Methods

### Study design and patients

This was a multicenter, retrospective study performed in 4 Spanish centers from April 2016 to November 2017. The study was approved by the Institutional Review Board at each hospital (CEIC del Hospital Clínico San Carlos de Madrid, Comité de Ética de la Investigación Provincial de Málaga y CEIC Hospital Vall d’Hebron) and was conducted in accordance with the principles of the Declaration of Helsinki. Prior to the analysis, all patients signed informed consent accepting the use of their blood samples stored in the Biobank for research purposes. Data from adult patients with a diagnosis of mCRC were included in the analysis if KRAS mutation status was determined in plasma both by OncoBEAM and Idylla kits and OncoBEAM KRAS-MUT positive results were <5% MAF. Patients having surgery of primary tumor or metastasectomy or that received chemotherapy/biologic agents <2 months prior the blood draw were excluded.

### Procedures

Blood was collected in either Streck BCT or EDTA tubes and processed into plasma using methods suitable for testing by both the OncoBEAM RAS CRC assay (3 mL) and the Idylla ctKRAS test (1 mL). Plasma samples were tested with OncoBEAM RAS CRC and *KRAS* mutant allele fraction was determined according to the kit’s instructions for use in those samples positive for *KRAS* mutation (KRAS-MUT+). Idylla testing was performed following the instructions and positive percent agreement (PPA) of KRAS-MUT+ results obtained by OncoBEAM and Idylla was calculated for patient samples having *KRAS* mutations at <5%, <1%, and <0.1% MAF by OncoBEAM. Due to the retrospective nature of the study, patient outcomes were available for a subset of patients and we determined the progression free survival (PFS) according to MAF values (1–5% vs <1%) in a cohort of 71 patients with at least 1 year of follow-up.

Finally, the clinical sensitivity of Idylla and OncoBEAM was evaluated by comparing plasma mutational analysis obtained by both methods on replicate samples to those obtained by standard-of-care (SOC) FFPE tumor KRAS (pyrosequencing, QIAGEN) testing on paired primary tumor specimens of KRAS-MUT+ patients with MAF values < 1% as determined by OncoBEAM.

### Statistical analysis

Categorical variables were summarized in numbers and percentages, continuous variables were presented as medians, minima and maxima. Comparison of detection thresholds was performed using the exact Mantel-Haenszel test. The PPA was also calculated. Paired proportions were compared using McNemar’s test. Relationships between MAF levels and clinical pathological characteristics were assessed using the Fisher exact test and the Student t-test. Median PFS was calculated by Kaplan-Meier estimation and compared with the log‐rank test. A p-value lower than 0.05 was considered statistically significant. Statistical analyses were performed using the statistical software SAS version 9.4.

## Data Availability

The datasets generated during and/or analysed during the current study are available from the corresponding author on reasonable request.
